# Mode differences in a mixed-mode health interview survey among adults

**DOI:** 10.1186/2049-3258-72-46

**Published:** 2014-12-22

**Authors:** Jens Hoebel, Elena von der Lippe, Cornelia Lange, Thomas Ziese

**Affiliations:** Department of Epidemiology and Health Monitoring, Robert Koch Institute, General-Pape-Straße 62-66, 12101 Berlin, Germany

**Keywords:** Health surveys, Data collection, Public health surveillance, Mixed-mode, Survey methods, Mode effects, Health indicators

## Abstract

**Background:**

Health interview surveys are important data sources for empirical research in public health. However, the diversity of methods applied, such as in the mode of data collection, make it difficult to compare results across surveys, time, or countries. The aim of this study was to explore whether the prevalence rates of health-related indicators amongst adults differ when self-administered paper mail questionnaires (SAQ-Paper), self-administered web surveys (SAQ-Web), and computer-assisted telephone interviews (CATI) are used for data collection in a health survey.

**Methods:**

Data were obtained from a population-based mixed-mode health interview survey of adults in Germany carried out within the ‘German Health Update’ (GEDA) study. Data were collected either by SAQ-Paper (n = 746), SAQ-Web (n = 414), or CATI (n = 411). Predictive margins from logistic regression models were used to estimate the prevalence rates of chronic conditions, subjective health, mental health, psychosocial factors, and health behaviours, adjusted for the socio-demographic characteristics of each mode group.

**Results:**

Socio-demographic characteristics were found to differ significantly between study participants who responded by SAQ-Paper, SAQ-Web, and CATI. Crude prevalence rates for health-related indicators also showed significant variation across all three survey modes. After adjusting for socio-demographic factors though, significant differences in prevalence rates between the two self-administered modes (SAQ-Paper and SAQ-Web) were found in only 2 out of the 19 health-related indicators studied. The differences between CATI and the two self-administered modes remained significant however, especially for indicators of mental and psychosocial health and self-reported sporting activity.

**Conclusions:**

The findings of this study indicate that prevalence rates obtained from health interview surveys can vary with the mode of data collection, primarily between interviewer and self-administered modes. Hence, the type of survey mode used should be considered when comparing results from different health surveys. Mixing self-administered modes, such as paper-based questionnaires and web surveys, may be a combination to minimize mode differences in mixed-mode health interview surveys.

**Electronic supplementary material:**

The online version of this article (doi:10.1186/2049-3258-72-46) contains supplementary material, which is available to authorized users.

## Background

Empirical research in public health is frequently based on data derived from population-based health interview surveys in which people of the general population are questioned about health issues. However, the methods applied, such as in the sampling procedures and modes of data collection, are diverse [1]. As a result, investigators face numerous challenges when attempting comparison of results across individual surveys, time, or countries.

When designing a survey, researchers aim to optimize data collection procedures and reduce total survey errors within available time and budget parameters [2]. Investigators have to find the most affordable and feasible survey methods, which can, however, differ across countries and settings or between different target populations. In certain cases, the best affordable method is a mixed-mode survey design in which different modes of data collection are offered to respondents [2]. Mixed-mode designs are considered to increase response rates, improve sample composition and data quality, and lower survey costs [3].

Nevertheless, different modes of data collection are known to have differing influences on the response behaviour of study participants. For instance, the amount of effort needed to answer a question can vary across modes and lead to a range of response errors referred to as ‘satisficing effects’ [4]. Another well-known and frequently described mode effect is the presence of an interviewer, the so called ‘interviewer effect’ [2, 5]. In personal interviews, such as face-to-face interviews (F2F), computer-assisted personal interviews (CAPI), or paper and pencil interviews (PAPI), the effects of social desirability can also be observed. This means that respondents will answer sensitive questions (for instance on drug consumption or sexual behaviours) in a way that fits societal norms, so as not to upset the interviewer or appear themselves to be deviant. Another mode effect is the tendency of respondents to agree with interviewer statements and answer positively to questions related to these (acquiescence), especially when interacting with another person [3]. Additionally, in the presence of an interviewer, respondents tend to round-up scalar or number questions (the heaping effect). A possible explanation for this particular behaviour is that respondents may feel time pressed because the interviewer is waiting for their answer and, thus, they allocate less time to remember or calculate exactly [6, 7].

Independently of the presence or absence of an interviewer, data collection methods can differ in two other important dimensions: how the information is presented (visual, oral, or both), and how the respondents convey their answers (spoken, written, or typed) [8, 9]. Previous research argues that in visual modes respondents tend to answer to categorical questions with the first categories (the primacy effect), while in oral modes respondents tend to choose the last categories (the recency effect) [10]. In oral modes, respondents also tend to give more positive answers on scale questions than do respondents in visual modes [3, 11]. Moreover, it is assumed that self-administered mode responses result in fuller use of the entire scale, while administered modes favour the end points [12]. As a whole, it is recognised that when modes differ at the two levels (e.g. interviewer and oral versus self-administered and visual), the quality varies more than when modes differ only at one level [13].

Mode effects have implications for the comparability of data collected by different survey modes. In recent years, a considerable number of studies have dealt with possible mode effects, their strength, and their impact on survey estimates. Unfortunately, it is not that easy to compare their results as most studies examined different populations and topics, applied different sampling procedures, and tested different questions and instruments. This may be one possible reason why many of the results are inconsistent and partially contradictory. Even within one study conclusions could sometimes not be drawn, as for some measures the mode of data collection may have had a strong effect, whereas for others, there was minimal evidence of mode effects [14].

Studies that compared web and paper-based questionnaires have found either very few mode effects, or none at all. Bäckström and Nilsson [15] found that the most prominent differences are related to the gender effect in web questionnaires. De Bernando and Curtis [16] report, though, that there are no significant mode differences once demographic variables (such as employment and income) are added to the analyses. Other studies have revealed that there is a higher response from the highly educated in web questionnaires, but that this does not affect the response behaviour of the participants [17]. McCabe et al. [18] did not find substantial differences in estimates of alcohol consumption between web and paper-based questionnaires in a non-randomised mixed-mode study.

Mode effects are more often found when comparing computer-assisted telephone interviewing (CATI) and web questionnaires. One consistent result is that people interviewed by telephone tend to give more positive answers to scale questions than people completing web questionnaires [19–21]. Positive answers to questions on the mental dimensions of health-related quality of life were also found to be higher in telephone surveys [22].

Substantial mode differences in the reporting of self-assessed health items are also shown in comparisons between CATI and self-administered paper questionnaires, where extreme response categories are used more frequently among telephone respondents (extreme response style) [23]. The authors therefore suggest caution when comparing prevalence rates across surveys or when studying time trends, as mode effects may be as large as the effects under investigation. A study of cannabis consumption of adults in Germany showed that there was a lower prevalence among interviewed people by telephone than among those who participated through paper-based questionnaires [24]. Another study on mode effects in health surveys revealed that in comparing face-to-face and self-administered modes there were no significant mode effects for indicators related to the use of health services, but there were significant mode effects for indicators related to self-reported health-related quality of life, health behaviour, social relations and morbidity [25].

In general, the greatest differences between modes are attributed to interviewer effects, especially on sensitive topics. When a set of modes is compared, it is relatively frequently reported that the largest mode effects can be observed between self-administered and personal interview modes, rather than within modes [21, 26, 27]. Interviewer effects are most often reported on the desirable or undesirable aspects of certain societal behaviours [12, 28, 29]. Although it is also reported that mode effects exist, and a face-to-face survey mode generates more socially desirable responses than a web survey, it also has to be recognized that those effects may not be as pervasive as might be expected [30].

The aim of this study was to explore whether the prevalence rates of certain health-related indicators are affected by differences in the types of survey modes used for data collection in a health survey of adults. A comparison of the prevalence rates of chronic conditions, subjective health, psychosocial factors, mental health, and health behaviours, adjusted for socio-demographic differences between mode groups, revealed by paper (mailed) questionnaires, web surveys and telephone interviews was carried out. The results of this study will contribute to a better understanding of the differences in the results of population-based health surveys that use different modes of data collection.

## Methods

### Material and study design

Data were obtained from a pilot study carried out within the ‘German Health Update’ (GEDA), a national health interview survey among adults in Germany. The GEDA study is part of the nationwide Health Monitoring System administered by the Robert Koch Institute, the national public health institute in Germany. The aim of the regularly conducted cross-sectional GEDA surveys is to provide current data on population health, health determinants, and the use of health services. Data are used for national and European Union health reporting, health policies and public health research [31, 32]. Previous GEDA surveys were designed as single-mode telephone surveys in which data were collected by CATI. Those surveys were based on samples of telephone numbers from the entire German fixed-line network. In view of increasing non-response errors and selection bias, a mixed-mode pilot study (GEDA 2.0) was carried out using a sample of addresses derived from local registry offices instead of a sample of telephone numbers. The aims of this pilot study were (1) to compare two mixed-mode survey designs with a single-mode telephone design in respect of response rates, sample compositions, and data quality, and (2) to explore whether estimates of health indicators differ between different modes of data collection. In the present paper, the focus is on the second aim of the study. Accordingly, data from GEDA 2.0 were used to investigate differences in prevalence rates between the three modes of data collection used in the study. It has to be acknowledged that the GEDA 2.0 study did not have an experimental design specifically tailored to the investigation of pure mode effects; nevertheless the data obtained in GEDA 2.0 allow for comparisons of health indicators between the different modes used. This can be achieved by statistical adjustment for socio-demographic differences between the mode groups due to differential non-response, similar to what was done in previous studies of mode differences [3, 16, 24, 25].

The GEDA 2.0 study was a pilot survey based on a sample of adults registered in the local resident registries of six municipalities covering urban and rural localities as well as the eastern and western regions of Germany. Subjects were selected using a disproportionate stratified random sampling procedure. A gross sample of 10,080 subjects was randomly allocated to three different designs: a) a sequential mixed-mode survey design; b) a simultaneous mixed-mode survey design; and c) a single-mode survey design. All selected subjects were invited by mail to participate. Data were collected by means of a self-administered web-based questionnaire (SAQ-Web), a self-administered mail questionnaire (SAQ-Paper), and a standardised computer-assisted telephone interview (CATI). In the sequential mixed-mode design, these three modes of data collection were offered step by step. First, subjects were invited to participate via web. If they did not answer, they were additionally offered a mail questionnaire. If they did not answer again, they were additionally asked to send their telephone number by mail for participating via CATI. In the simultaneous mixed-mode design, all three modes were offered to respondents at once and they could choose the one they preferred. Finally, those subjects allocated to the single-mode design could only participate via CATI.

A total of 1,571 respondents completed the GEDA 2.0 survey between August and November 2012. In the sequential mixed-mode design, 290 participants (51.7%) responded via SAQ-Web, 264 participants (47.1%) used SAQ-Paper, and 7 participants (1.3%) were interviewed via CATI. In the simultaneous mixed-mode design, 124 participants (20.1%) chose the SAQ-Web option, 482 participants (78.1%) responded by SAQ-Paper, and 11 participants (1,8%) chose CATI. In the single-mode design, a total of 393 CATI interviews were carried out. For the present study, data from all three survey designs were pooled to analyse differences in prevalence rates between the three modes of data collection. Due to the very low number of CATI participants in the mixed-mode designs and the very unequal number of SAQ-Web and SAQ-Paper participants in the mixed-mode designs, mode differences in prevalence rates by survey design could not be examined.

According to the internationally used AAPOR Standard Definitions of outcome rates for surveys [33], the ‘Response Rate 1’ for all three survey designs together was 16.3%. This response rate, also known as the AAPOR minimum response rate, is the number of complete interviews divided by the number of interviews plus the number of non-interviews plus all cases of unknown eligibility.

The questionnaires contained questions on health and diseases, health behaviour, and socio-demographic characteristics. The wording and order of questions and answers did not differ between modes. The study was approved by The Federal Commissioner for Data Protection and Freedom of Information. Informed consent was obtained from all participants in advance.

### Health indicators

We compiled a set of health-related indicators for examining mode differences. Respondents were asked whether they had ever been diagnosed by a doctor as having had diabetes, hypertension, dyslipidaemia, coronary heart disease, chronic bronchitis, bronchial asthma, and/or osteoarthrosis. Respondents who answered ‘yes’ were asked whether they had been suffering from this disease during the past 12 months. The prevalence of obesity was assessed by calculating body mass index (BMI) using the World Health Organization criteria (BMI ≥ 30 kg/m^2^) [34]. We derived BMI from respondent’s self-reported height and weight. Subjective health was assessed with the Minimum European Health Module (MEHM) including questions on self-rated health, chronic conditions, and long-term activity limitations [35].

Depressive symptoms were measured using the eight-item Patient Health Questionnaire depression scale (PHQ-8) [36]. Respondents were asked about the presence and frequency of depressive symptoms over the last two weeks (response categories: ‘not at all’, ‘several days’, ‘more than half the days’, or ‘nearly every day’). Current depression was assessed using the diagnostic algorithm for PHQ-8 [36]. Additionally, one item of the Budapest Initiative Mark 2 questionnaire (BI-M2) was used to look at self-reports on mental health [37]. Participants were asked how often they felt depressed (‘daily’, ‘weekly’, ‘monthly’, ‘a few times a year’, or ‘never’). We then used the five-item WHO Well-Being Index (WHO-5) to measure current mental well-being [38]. This instrument consists of five questions about the frequency of happiness, calm, having energy, feeling fresh, and interest in daily things during the past two weeks. We considered the positive category ‘very good or excellent well-being’ (sum score > 75) and the negative category ‘poor or minimal well-being’ (sum score ≤ 25) in this analysis. Perceived social support was measured using the three-item Oslo Social Support scale (OSS-3) [39]. Respondents were asked about the number of people they could count on, the level of other people’s interest in their lives, and the availability of help from neighbours. We distinguished between ‘poor’, ‘intermediate’, and ‘strong’ social support [40]. We also used information on personal health behaviours, such as current smoking status, alcohol consumption [41, 42], physical activity [43], sporting activity in the last three months, and participation in vaccination programmes. For the most of the described health indicators, the percentage of missing values differed significantly between the modes of data collection, usually with the highest rate of missing values in the SAQ-Paper mode [see Additional file 1].

### Socio-demographic characteristics

A range of socio-demographic characteristics was considered in the analyses. Age at time of interview was calculated using information on year and month of birth. Educational level was measured using the ‘Comparative Analysis of Social Mobility in Industrial Nations’ (CASMIN) [44]. Income level was assessed by a question on monthly household net income, and income quintiles were calculated. Furthermore, we included information on current employment status, marital status, and type of household.

### Statistical analysis

As a first step, we calculated crude prevalence rates of diagnosed physical conditions, subjective health, depression, mental well-being, social support, and health behaviours by mode of data collection. Pearson’s χ^2^-tests were used to examine for statistically significant differences (α = 0.05). Second, we adjusted these prevalence rates for socio-demographic factors to investigate whether mode differences in crude prevalence rates can be accounted for by socio-demographic differences between the mode groups. Socio-demographically adjusted prevalence rates were then calculated using predictive margins [45] computed on the basis of logistic regression models containing socio-demographic factors (age, gender, education, income, labour status, marital status, type of household) and the mode of data collection as covariates. These predictive margins (also called ‘predicted marginal proportions’) represent the weighted average of the predicted probabilities of a respective health-related outcome in each mode group. We used z-tests to examine for statistically significant differences. Previous studies suggest that socio-demographic measures are widely mode-insensitive [22]. Hence, the socio-demographic measures used were assumed to be suitable for the adjustment of known socio-demographic differences between the three mode groups.

## Results and discussion

### Sample characteristics

Respondents who participated by SAQ-Paper, SAQ-Web, and CATI differed significantly in their socio-demographic characteristics (Table 1). SAQ-Web participants were younger, had higher education levels, higher incomes, and higher labour market participation rates, and were more likely to be single and to live in a multi-person household than those who responded by SAQ-Paper and CATI modes. The gender ratio was more balanced in SAQ-Web than in SAQ-Paper and CATI. Participants who responded by SAQ-Paper were less educated and had lower incomes than those who responded via CATI or SAQ-Web. More than a third of the people who participated through SAQ-Paper or CATI modes were aged 65 or above.

**Table 1:** **Socio-demographic characteristics of the mode groups in the sample (GEDA 2.0 pilot study, Germany, August – November 2012)**

	SAQ-Paper (n = 746)		SAQ-Web (n = 414)		CATI (n = 411)		
	n	%	n	%	n	%	p-value
**Gender**							
women	448	60.0	202	48.8	230	56.0	<0.01
men	298	40.0	212	51.2	181	44.0	
**Age**							
18-29 years	96	12.9	101	24.4	53	12.9	<0.001
30-44 years	141	18.9	121	29.2	83	20.2	
45-64 years	231	31.0	134	32.4	129	31.4	
65+ years	278	37.3	58	14.0	146	35.5	
**Education**							
low	187	25.3	48	11.6	65	15.8	<0.001
medium	380	51.4	213	51.5	196	47.7	
high	173	23.4	153	37.0	150	36.5	
**Household net income**							
quintile 1 (low)	174	24.6	58	14.8	63	16.8	<0.001
quintile 2	170	24.1	71	18.1	66	17.6	
quintile 3	155	21.9	66	16.8	81	21.6	
quintile 4	117	16.6	93	23.7	89	23.7	
quintile 5 (high)	91	12.9	104	26.5	76	20.3	
**Labour market status**							
working	353	50.0	313	75.6	222	54.1	<0.001
non-working	353	50.0	101	24.4	188	45.9	
**Marital status**							
single	157	21.4	136	32.9	95	23.1	<0.001
separated/divorced/widowed	131	17.9	36	8.7	76	18.5	
married and cohabiting	445	60.7	242	58.5	240	58.4	
**Type of household**							
one-person household	159	21.8	65	15.8	75	18.3	<0.05
multi-person household	571	78.2	346	84.2	335	81.7	

SAQ-Paper = self-administered paper mail questionnaire; SAQ-Web = self-administered web survey; CATI = computer-assisted telephone interview.

### Physical conditions and subjective general health

Table 2 shows the prevalence of health-related physical conditions by mode. Most of the crude disease prevalence rates were significantly lower for SAQ-Web than for SAQ-Paper and CATI respondents. Adjustment for socio-demographic characteristics of the mode groups appreciably altered the patterns of prevalence rates. With regard to the 12-month prevalence rates of diseases, we did not find any mode differences after adjusting for socio-demographic factors. However, in respect of lifetime prevalence rates, differences in respiratory diseases (chronic bronchitis and bronchial asthma) between the telephone mode and self-administered modes remained significant.

**Table 2:** **Crude and socio-demographic-adjusted prevalence rates of physical conditions and subjective health measures by mode (GEDA 2.0 pilot study, Germany, August – November 2012)**

	Crude prevalence						Adjusted prevalence*					
	SAQ-Paper	SAQ-Web	CATI	SAQ-Paper vs. SAQ-Web	SAQ-Paper vs. CATI	SAQ-Web vs. CATI	SAQ-Paper	SAQ-Web	CATI	SAQ-Paper vs. SAQ-Web	SAQ-Paper vs. CATI	SAQ-Web vs. CATI
	%	%	%	p-value	p-value	p-value	%	%	%	p-value	p-value	p-value
***Physical conditions***												
**Diagnosed diabetes**												
12-month-prevalence	11.1	4.8	10.1	<0.001	n.s.	<0.01	9.3	7.1	8.9	n.s.	n.s.	n.s.
**Diagnosed hypertension**												
12-month prevalence	36.6	22.0	33.1	<0.001	n.s.	<0.001	31.6	29.2	29.8	n.s.	n.s.	n.s.
**Diagnosed dyslipidaemia**												
12-month prevalence	25.9	15.7	23.0	<0.001	n.s.	<0.01	21.4	23.2	20.4	n.s.	n.s.	n.s.
**Obesity (BMI ≥ 30)**												
Point prevalence	20.9	15.2	16.8	<0.05	n.s.	n.s.	19.5	18.7	16.3	n.s.	n.s.	n.s.
**Diagnosed CHD**												
Lifetime prevalence	9.0	3.4	9.9	<0.001	n.s.	<0.001	6.9	6.3	9.3	n.s.	n.s.	n.s.
**Diagnosed chronic bronchitis**												
Lifetime prevalence	8.8	4.6	11.2	<0.01	n.s.	<0.001	7.7	4.9	10.3	n.s.	n.s.	<0.01
**Diagnosed bronchial asthma**												
Lifetime prevalence	10.3	9.2	16.6	n.s.	<0.01	<0.01	10.0	9.2	16.0	n.s.	<0.01	<0.01
**Diagnosed osteoarthrosis**												
Lifetime prevalence	29.8	14.7	24.9	<0.001	n.s.	<0.001	25.3	21.1	21.6	n.s.	n.s.	n.s.
***Subjective health***												
**MEHM: Self-rated health**												
Very good	11.0	18.8	18.3	<0.001	<0.01	n.s.	13.2	14.8	19.0	n.s.	<0.05	n.s.
Poor/very poor	4.1	2.7	4.9	n.s.	n.s.	n.s.	3.4	4.4	4.2	n.s.	n.s.	n.s.
**MEHM: Chronic health problem**												
Yes	44.2	32.2	44.5	<0.001	n.s.	<0.001	40.0	39.4	42.3	n.s.	n.s.	n.s.
**MEHM: Daily activity limitations**												
Yes	37.5	21.8	33.1	<0.001	n.s.	<0.001	33.4	28.1	29.9	n.s.	n.s.	n.s.

SAQ-Paper = self-administered paper mail questionnaire; SAQ-Web = self-administered web survey; CATI = computer-assisted telephone interview.

*adjusted for age, gender, education, income, labour status, marital status, type of household.

CHD = coronary heart disease.

MEHM = Minimum European Health Module.

n.s. = not significant (p > 0.05); vs. = versus.

The crude prevalence rates for subjective health indicators showed a higher self-rating for health by SAQ-Web participants compared with that by SAQ-Paper and CATI participants. However, after adjusting for socio-demographic characteristics no statistically significant differences between the two self-administered modes were evident. Participants interviewed by telephone rated their health more frequently as ‘very good’ (positive extreme category) than respondents who participated via SAQ-Paper.

The results indicate that any variations in the prevalence rates of particular physical conditions and self-rated global health across survey modes can largely be explained by socio-demographic differences between the groups of respondents. No differences between the two self-administered modes were found after adjustment for socio-demographic factors. These findings support previous research suggesting that self-reports on global health do not vary between the different kinds of self-administered modes [16]. However, Shim et al. [46] found that web respondents report better self-rated health than SAQ-Paper respondents. Because of this inconsistency, further research on differences in reports on global health between paper-based and web-based questionnaires is required. Other studies have indicated that people rate their health more highly in telephone surveys than they do in self-administered surveys [23, 47–49]. This is supported by our findings, but not every item of subjective health seems to be affected. The differences in self-reports of respiratory diseases between interviewer and self-administered modes indicated by the present findings have not been found in a comparison of face-to-face and self-administered modes [25]. Therefore, future studies should scrutinise potential mode differences in self-reports about chronic conditions.

### Mental and psychosocial health

Prevalence rates relating to depression, mental well-being, and social support are presented in Table 3. The crude prevalence of current depression as defined by the PHQ-8 diagnostic algorithm was higher for CATI-based respondents than for those using SAQ-Web, while after adjusting for socio-demographic characteristics no significant mode difference for depression persisted. The subjective indicator ‘feeling depressed’ showed higher prevalence rates of the positive extreme category ‘never’ in the CATI interview mode compared with the self-administered modes when socio-demographic variables were controlled for. With regard to mental well-being, the crude prevalence of poor or minimal well-being differed significantly across modes. After adjustment for socio-demographic characteristics, the lower percentage of poor or minimal well-being in CATI administered surveys compared with those using SAQ-Paper remained significant. With regard to the positive category (very good or excellent well-being) no significant mode differences were observed. Social support was found to be strongest in CATI and poorest in SAQ-Paper. Large differences in social support between all three survey modes also persisted after adjustment for socio-demographic characteristics.

**Table 3:** **Crude and socio-demographic-adjusted prevalence rates of depression, mental well-being, and social support by mode (GEDA 2.0 pilot study, Germany, August – November 2012)**

	Crude prevalence						Adjusted prevalence*					
	SAQ-Paper	SAQ-Web	CATI	SAQ-Paper vs. SAQ-Web	SAQ-Paper vs. CATI	SAQ-Web vs. CATI	SAQ-Paper	SAQ-Web	CATI	SAQ-Paper vs. SAQ-Web	SAQ-Paper vs. CATI	SAQ-Web vs. CATI
	%	%	%	p-value	p-value	p-value	%	%	%	p-value	p-value	p-value
**PHQ-8: diagnostic algorithm**												
Current depression	12.8	10.1	15.3	n.s.	n.s.	<0.05	12.0	11.0	15.3	n.s.	n.s.	n.s.
**BI-M2: Feeling depressed**												
Never	35.0	27.3	40.1	<0.01	n.s.	<0.001	33.0	28.4	39.2	n.s.	<0.05	<0.01
Daily/weekly	13.5	12.6	10.8	n.s.	n.s.	n.s.	13.4	13.6	11.0	n.s.	n.s.	n.s.
**WHO-5: Well-being score (0–100)**												
Very good or excellent (score > 75)	28.2	27.3	33.5	n.s.	n.s.	n.s.	27.1	30.6	32.4	n.s.	n.s.	n.s.
Poor or minimal (score ≤ 25)	13.5	7.5	4.9	<0.01	<0.001	n.s.	11.8	8.2	4.9	n.s.	<0.001	n.s.
**OSS-3: social support score (3–14)**												
Strong social support (score ≥ 12)	23.2	31.8	39.3	<0.01	<0.001	<0.05	24.2	30.2	40.2	<0.05	<0.001	<0.01
Poor social support (score ≤ 8)	21.0	15.0	11.1	<0.05	<0.001	n.s.	19.0	17.0	11.0	n.s.	<0.001	<0.05

SAQ-Paper = self-administered paper mail questionnaire; SAQ-Web = self-administered web survey; CATI = computer-assisted telephone interview.

PHQ-8 = Eight-item Patient Health Questionnaire depression scale.

BI-M2 = Budapest Initiative Mark 2 questionnaire.

WHO-5 = Five-item WHO Well-Being Index.

OSS-3 = Three-item Oslo Social Support scale.

*adjusted for age, gender, education, income, labour status, marital status, type of household.

n.s. = not significant (p > 0.05); vs. = versus.

These results indicate that respondents surveyed by an interviewer rate their mental and psychosocial health as being better than respondents who participate via self-administered modes. The positive patterns found in telephone surveys are also apparent in questions related to the mental dimensions of people’s health related quality of life or depression and stress [22, 23]. The most favoured explanations for these differences is that the interview respondents might be subject to social desirability bias and may seek to answer the questions in a manner that will be viewed favourably by the interviewer [25]. Studies comparing other forms of modes also report differences in mental health syndrome when an interviewer is involved [50], and no differences when diverse self-administered modes are compared [51].

Similarly, the differences in the social support answers could be also to some extent be explained by a social desirability effect. However, the differences in strong social support between SAQ-Paper and SAQ-Web respondents may have other explanations. There could be influence from additional circumstances which we could not account for. Further research is necessary to identify the real reasons for the differences between SAQ-Paper and SAQ-Web answers, as inconsistencies in the results concerning social relations have also been found in other studies [25, 52].

### Health behaviours

The prevalence of health behaviours is shown in Table 4. Smoking did not differ by mode, either before or after adjusting for socio-demographic variables. The crude prevalence of people never drinking alcohol was lower for the SAQ-Web respondents than for CATI respondents, while the socio-demographic-adjusted prevalence rates showed no statistically significant differences in drinking patterns across modes. With respect to the crude prevalence of physical activity measure, there were no significant differences between SAQ-Paper and SAQ-Web respondents, while there were significant differences between CATI and SAQ-Paper as well as CATI and SAQ-Web respondents. After adjusting for socio-demographic characteristics, all the significant differences disappeared except for the difference in high physical activity between CATI and SAQ-Web. High sporting activity was reported much more frequently by CATI respondents than by those respondents who participated via SAQ-Web and SAQ-Paper (differences between SAQ-Web and SAQ-Paper were not observed). The prevalence of no sporting activity was in turn higher for SAQ-Paper than for SAQ-Web and CATI respondents. These distinct differences remained significant after adjustment for socio-demographic factors. Conversely, differences in participation in influenza vaccination programmes were only observed in the crude prevalence rates, while the adjusted percentages showed no statistically significant variation across modes. Altogether, these results show that for the majority of the health-related behaviours there are no mode differences. Regarding sporting activity though, researchers should be cautious when interpreting data collected by different modes.

**Table 4:** **Crude and socio-demographic-adjusted rates of positive and negative responses on health behaviour measures by mode. (GEDA 2.0 pilot study, Germany, August – November 2012)**

	Crude prevalence						Adjusted prevalence*					
	SAQ-Paper	SAQ-Web	CATI	SAQ-Paper vs. SAQ-Web	SAQ-Paper vs. CATI	SAQ-Web vs. CATI	SAQ-Paper	SAQ-Web	CATI	SAQ-Paper vs. SAQ-Web	SAQ-Paper vs. CATI	SAQ-Web vs. CATI
	%	%	%	p-value	p-value	p-value	%	%	%	p-value	p-value	p-value
**Smoking**												
Daily	18.6	16.2	16.1	n.s.	n.s.	n.s.	18.0	15.2	17.5	n.s.	n.s.	n.s.
Never	46.1	44.4	43.6	n.s.	n.s.	n.s.	45.4	46.0	42.6	n.s.	n.s.	n.s.
**AUDIT-C: Alcohol consumption**												
Hazardous consumption	29.2	33.3	30.7	n.s.	n.s.	n.s.	31.2	31.0	31.2	n.s.	n.s.	n.s.
Never-drinkers	12.4	8.9	13.3	n.s.	n.s.	<0.05	10.9	10.9	13.3	n.s.	n.s.	n.s.
**Physical activity**												
≥5 days a week ≥30 minutes a day	16.8	15.5	21.7	n.s.	<0.05	<0.05	16.4	15.5	21.4	n.s.	n.s.	<0.05
<2.5 h a week	62.2	63.2	55.9	n.s.	<0.05	<0.05	62.2	63.2	56.5	n.s.	n.s.	n.s.
**Sporting activity**												
More than four times a week	17.8	17.3	30.2	n.s.	<0.001	<0.001	16.8	18.6	30.4	n.s.	<0.001	<0.001
None	25.1	15.5	16.8	<0.001	<0.01	n.s.	23.0	16.9	17.0	<0.05	<0.05	n.s.
**Participation in influenza vaccination** ^**#**^												
Yes	38.1	25.2	41.1	<0.001	n.s.	<0.001	33.7	32.3	38.2	n.s.	n.s.	n.s.

SAQ-Paper = self-administered paper mail questionnaire; SAQ-Web = self-administered web survey; CATI = computer-assisted telephone interview.

*adjusted for age, gender, education, income, labour status, marital status, type of household.

AUDIT-C = Alcohol Use Disorders Identification Test - Consumption.

^#^during the past winter season (2011/2012).

n.s. = not significant (p > 0.05); vs. = versus.

The results described here are to some extent comparable with findings from other studies. It was previously suggested that there are no differences between self-administered and interviewer modes according to smoking rates [23, 25] and alcohol consumption [18, 25]. A more detailed investigation, however, showed that while there are no differences in the number of people smoking and in alcohol consumption, there are differences in the level of consumption – the number of cigarettes smoked per day and the number of units of alcohol consumed [52]. There are also studies that have reported mode effects in alcohol consumption measures, with self-administered modes showing a higher rate of consumption compared with that of modes involving an interviewer [53, 54].

Studies that have looked at measures of physical activity also find differences according to survey mode. Modes in which an interviewer is involved show higher levels of physical activity than self-administered modes [25, 52]. This is consistent with our findings on physical activity, and partly on sporting activity. However, the differences between SAQ-Paper and SAQ-Web respondents in the category of no sporting activity might have another explanation. It is possible that there are some characteristics of the respondents that we have not accounted for in the adjusted models, such as their occupations. For instance, people who have an occupation which requires a lot of physical activity, normally, are less active in their leisure time [55]. People with such occupation possibly tend to participate in surveys using paper-based questionnaire rather than web-based questionnaires. This would explain the lower level of sporting activity among participants in SAQ-Paper compared with the SAQ-Web mode.

### Strength and limitations

The strength of this study was that the respondents who participated via SAQ-Paper, SAQ-Web, and CATI were selected from an identical sampling frame. Moreover, all respondents were surveyed in the same time period and in the same regions of Germany. The contents of the questionnaires, the wording of questions and response categories, as well as the order of questions in the questionnaire were also identical in each mode. In spite of these strengths, there are several limitations to our study. The pilot study GEDA 2.0 was predominantly designed to compare two mixed-mode survey designs with one single mode survey design in respect of response rates and sample composition. Therefore, subjects were randomly allocated to three survey designs but not to the three modes of data collection. The present study on mode differences was, hence, a secondary analysis of the data obtained in the GEDA 2.0 study. Considering the study design, it has to be acknowledged that the identified differences in health indicators across survey modes might not solely be caused by the effects of mode type on response patterns. Because of selection effects arising from mode-specific non-response, there may also be differences between the three mode groups according to characteristics of the respondents that were not measured in the survey. As a consequence, the identified mode differences in health indicators might also be caused by composition effects, which probably could not be completely disentangled from the influence of mode effects by statistical adjustment for known socio-demographic differences. Furthermore, it has to be acknowledged that smaller differences between modes might not have been detected due to a lack of statistical power. Additionally, for the latter reason we did not investigate whether mode differences in prevalence rates vary by age group, gender, or educational level. Therefore, future research should examine whether mode differences are moderated by socio-demographic or other characteristics. In addition, it should be borne in mind that the results of this study are based on a cross-sectional survey. Probably, the mode differences found here cannot simply be transferred to longitudinal surveys. Another issue to be considered is that we had to combine two response categories of certain health indicators (feeling depressed: ‘daily’/‘weekly’, self-rated health: ‘poor’/’very poor’) due to low case numbers. This may have masked potential differences between modes.

Due to the relatively low response rate, the results of this study may be affected by selection bias. A possible explanation for the low response rate may be that those subjects allocated to the single-mode survey design (1/3 of the gross sample), in which solely CATI was offered, were asked to send their phone number by post in advance of the telephone interview. This additional effort and transfer of personal data may have substantially lowered the willingness to participate in the study. A possible selection bias due to this should be borne in mind when interpreting the findings.

## Conclusions

In summary, the findings of this study indicate that prevalence rates obtained from health interview surveys can vary with the mode of data collection used in the survey. However, objective indicators based on factual issues, such as questions on prevalent diseases, may be less affected than subjective indicators of psychosocial and mental health, or health behaviours. Therefore, the mode of data collection should be considered when comparing results from different health interview surveys, or when the survey mode in periodically conducted surveys is changed over time. Moreover, our findings suggest that mode differences mainly exist between interviewer modes and self-administered modes, rather than between different kinds of self-administered questionnaires. Consequently, mixing self-administered modes, such as SAQ-Paper and SAQ-Web, may be a combination to minimize mode differences in mixed-mode health interview surveys [8]. However, the mode of data collection is only one among many factors that contribute to the total error of an estimate derived from a sample survey [56, 57]. The decision to use a mixed-mode design may depend on wider issues; such as the target population under study, the available time and budget for the study, or the questions to be asked in the survey [2].

## Electronic supplementary material

Additional file 1: Missing values for health indicators by mode of data collection.(PDF 206 KB)
