# Supplementary material for: Mode differences in a mixed-mode health interview survey among adults
Source: Arch Public Health. 2014 Dec 22;72:46. doi: 10.1186/2049-3258-72-46 (PMC4373057; doi:10.1186/2049-3258-72-46)
Supplement: Supplementary file 1 — Additional file 1: Missing values for health indicators by mode of data collection.(PDF 206 KB) [file 13690_2014_5065_MOESM1_ESM.pdf]

Additional file 1 – Missing values for health indicators by mode of data collection

Table A1 - Percentage of missing values for health indicators by mode (GEDA 2.0 pilot study, Germany, August – November 2012)

	SAQ-Paper (n=746)	SAQ-Web (n=414)	CATI (n=411)	SAQ-Paper vs. SAQ-Web	SAQ-Paper vs. CATI	SAQ-Web vs. CATI
	%	%	%	p-value	p-value	p-value
**Physical conditions**						
Diagnosed diabetes	7.2	0.0	0.7	<0.001	<0.001	n.s.
Diagnosed hypertension	7.8	0.0	0.7	<0.001	<0.001	n.s.
Diagnosed dyslipidaemia	7.8	0.0	0.7	<0.001	<0.001	n.s.
Obesity (Body mass index ≥ 30)	3.0	1.5	1.7	n.s.	n.s.	n.s.
Diagnosed coronary heart disease	1.5	0.0	1.2	<0.05	n.s.	<0.05
Diagnosed chronic bronchitis	7.0	0.2	0.2	<0.001	<0.001	n.s.
Diagnosed bronchial asthma	7.4	0.0	0.2	<0.001	<0.001	n.s.
Diagnosed osteoarthrosis	6.8	0.0	1.2	<0.001	<0.001	<0.05
**Subjective health**						
MEHM: Self-rated health	1.1	0.0	0.0	<0.05	<0.05	-
MEHM: Chronic health problem	2.0	2.4	0.0	n.s.	<0.001	<0.01
MEHM: Daily activity limitations	3.9	3.4	0.0	n.s.	<0.001	<0.001
**Psychological/psychosocial health**						
PHQ-8: current depression	9.0	4.4	1.2	<0.01	<0.001	<0.01
WHO-5: mental well-being score	6.4	3.4	1.2	<0.05	<0.001	<0.05
OSS-3: social support score	2.4	0.0	1.5	<0.001	n.s.	<0.05
**Health behaviours**						
Smoking	3.2	0.0	0.0	<0.001	<0.001	-
AUDIT-C: Alcohol consumption	11.3	1.9	1.0	<0.001	<0.001	n.s.
Sporting activity	2.1	0.0	0.0	<0.01	<0.01	-
Participation in influenza vaccination	3.9	0.2	0.0	<0.001	<0.001	n.s.

SAQ-Paper = self-administered paper mail questionnaire; SAQ-Web = self-administered web survey; CATI = computer-assisted telephone interview; MEHM = Minimum European Health Module; PHQ-8 = Eight-item Patient Health Questionnaire depression scale; BI-M2 = Budapest Initiative Mark 2 questionnaire; WHO-5 = Five-item WHO Well-Being Index; OSS-3 = Three-item Oslo Social Support scale; AUDIT-C = Alcohol Use Disorders Identification Test–Consumption; n.s. = not significant (p>0.05); vs. = versus
